# The Effect of Dia2 Protein Deficiency on the Cell Cycle, Cell Size, and Recruitment of Ctf4 Protein in *Saccharomyces cerevisiae*

**DOI:** 10.3390/molecules27010097

**Published:** 2021-12-24

**Authors:** Aneliya Ivanova, Aleksandar Atemin, Sonya Uzunova, Georgi Danovski, Radoslav Aleksandrov, Stoyno Stoynov, Marina Nedelcheva-Veleva

**Affiliations:** Laboratory of Genomic Stability, Institute of Molecular Biology, Bulgarian Academy of Sciences, Acad. G., Bonchev Str. Bl.21, 1113 Sofia, Bulgaria; anivanova@bio21.bas.bg (A.I.); atemin@bio21.bas.bg (A.A.); sonia_dimitrova84@abv.bg (S.U.); georgi_danovski@abv.bg (G.D.); raleksandrov@bio21.bas.bg (R.A.)

**Keywords:** DNA replication stress, DNA replication, DNA repair, cell cycle control, Dia2, Ctf4, cell size control

## Abstract

Cells have evolved elaborate mechanisms to regulate DNA replication machinery and cell cycles in response to DNA damage and replication stress in order to prevent genomic instability and cancer. The E3 ubiquitin ligase SCF^Dia2^ in *S. cerevisiae* is involved in the DNA replication and DNA damage stress response, but its effect on cell growth is still unclear. Here, we demonstrate that the absence of Dia2 prolongs the cell cycle by extending both S- and G2/M-phases while, at the same time, activating the S-phase checkpoint. In these conditions, Ctf4—an essential DNA replication protein and substrate of Dia2—prolongs its binding to the chromatin during the extended S- and G2/M-phases. Notably, the prolonged cell cycle when Dia2 is absent is accompanied by a marked increase in cell size. We found that while both DNA replication inhibition and an absence of Dia2 exerts effects on cell cycle duration and cell size, Dia2 deficiency leads to a much more profound increase in cell size and a substantially lesser effect on cell cycle duration compared to DNA replication inhibition. Our results suggest that the increased cell size in *dia2*∆ involves a complex mechanism in which the prolonged cell cycle is one of the driving forces.

## 1. Introduction

One of the essential tasks of every living cell is to duplicate its genome accurately. When faithful DNA replication is hindered, genome stability may be compromised, leading to cell cycle arrest or cell death. The accumulation of mutations in the genome caused by perturbed DNA replication is implicated in cellular senescence and may lead to multiple disorders, such as cancer and neurodegenerative diseases [1]. To precisely execute the dynamic program of the replication machinery and minimize the likelihood of replication errors, cells control both the activity and abundance of various DNA replication proteins tightly [2]. Two essential proteins that contribute to maintaining genome stability during DNA synthesis in *Saccharomyces cerevisiae* are Ctf4 (Mcl1 in *S. pombe* and And1 in *H. sapiens* [3]) and Dia2 (Pof3 in *S. pombe* [4]). And1/Ctf4 forms a homotrimer that binds to the proteins Mcm10, GINS, and Polα [5]. Ctf4 stabilizes chromatin-associated Polα onto chromatin [6,7] and is involved in sister-chromatid cohesion [8,9]. Although Ctf4 is not essential for budding yeast viability, the loss of Ctf4 leads to G2/M delay [10] and the activation of the S-phase checkpoint [11]. Both Ctf4 and And1 are required for efficient DNA replication [5,11], acting as a hub that coordinates the Polα/Primase complex and the active replication helicase CMG (Cdc45-Mcm2-7-GINS) [12,13]. Ctf4 and heterotrimeric complex Csm3/Tof1/Mrc1 directly associate the CMG helicase and facilitate replisome progression [14], and are fundamental for keeping the integrity of the fork by harmonizing the replicative helicase [15] to DNA polymerases. In addition, Ctf4, together with Mrc1, is required for the stable association of the E3 ubiquitin ligase complex SCF^Dia2^ (named after its main components, Skp1, Cullin, and an F-box protein) with the replisome [16,17,18]. The F-box protein Dia2, one of 21 substrate recognition F-box proteins in *S. cerevisiae*, is involved in the regulation of cell cycle progression, DNA replication, and DNA damage stress response [19,20,21]. When the DIA2 gene is deleted, the S-phase checkpoint is activated, and cells retain in the S/G2/M-phase of the cell cycle and become hypersensitive to DNA damaging agents [19,20,21]. During DNA replication termination, the recruitment of Dia2 to replication forks leads to the ubiquitination of an Mcm7 subunit of the Mcm2-7 complex and the consecutive disassembly of the active CMG helicase [22]. Both Mrc1 and Ctf4 physically interact with Dia2, and this interaction is responsible for the destabilization of Mrc1 in a proteasome-dependent manner during S-phase checkpoint recovery [23,24]. In addition, it was demonstrated in vitro that both Mrc1 and Ctf4 are targets for ubiquitination during replication termination [25].

Interestingly, in DIA2 deletion strains (*dia2*∆), a fraction of the cell population undergoes slight elongation and filament formation [26,27]. This phenotype, known in *S. cerevisiae* as pseudohyphal growth, consists of chains of elongated, mononucleated cells that form a filament. The cells remain connected following cytokinesis and exhibit an altered cell cycle with an extended budding period. The regulation of pseudohyphal growth is complex, but it is known that the Flo11 protein, a cell-surface flocculin required for the formation of connections between cells, is up-regulated [26].

It is still unclear how Ctf4 and Dia2 proteins cooperate in maintaining genomic stability and whether changes in cell morphology, as a result of DIA2 deletion, is related to its function in DNA replication and cell cycle progression. To approach this issue, we studied the cell phase duration, cell size, and recruitment of Ctf4 when the Dia2 protein is missing.

## 2. Results

### 2.1. Loss of Dia2 Protein Extends Cell Cycle Duration

**Figure 1:**
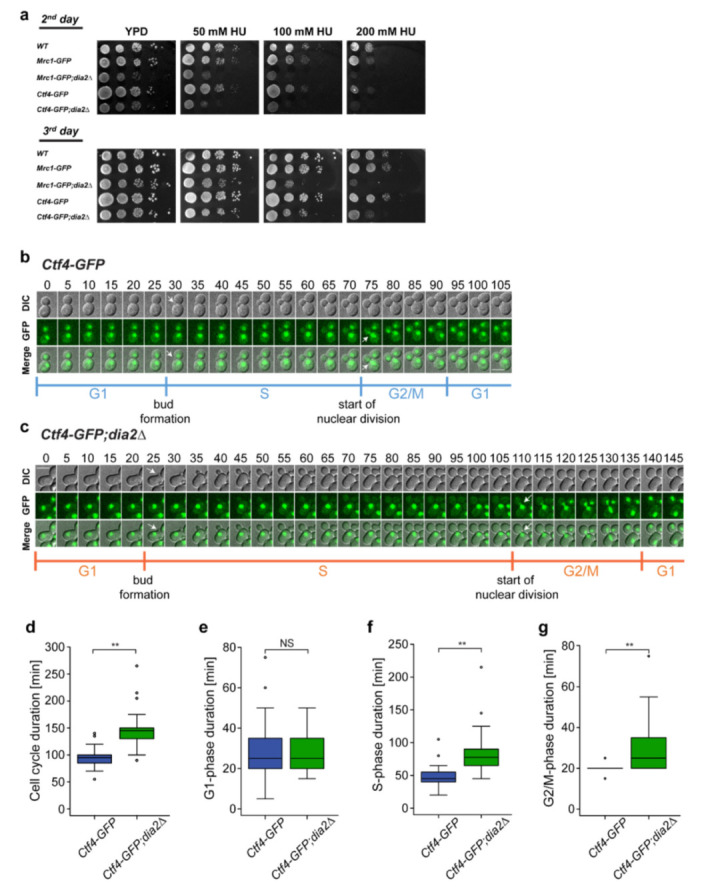
Dia2 deficiency increases the length of the cell cycle due to prolonged S- and G2/M-phases. (**a**) Viability test: *S. cerevisiae* cells from the *Ctf4-GFP*, *Ctf4-GFP;dia2*Δ, *Mrc1-GFP*, *Mrc1-GFP;dia2*Δ, and control wild-type S288C strains were tested by 10-fold serial dilution assay. A total of 5 μL of each dilution were spotted onto YPD or YPD supplemented with 50 mM, 100 mM, and 200 mM HU. The images represent cell growth on the second and third day of incubation. (**b**) Live-cell imaging of an entire cell cycle of a typical cell expressing nuclear localized Ctf4-GFP protein. Visualization of the whole cell and nucleus is obtained by DIC and GFP, respectively. Frames were taken every 5 min and maximum intensity projection of 11 z-planes was applied. Arrows indicate the onset of bud formation (G1-to-S transition) and the start of nuclear division (S-to-G2/M transition). The scale bar is 5 μm. (**c**) Live-cell imaging of an entire cell cycle of a typical cell from the *Ctf4-GFP;dia2*Δ represented in the same way as (**b**). (**d**–**g**) Mean value ± standard deviation of the duration of the entire cell cycle, G1-, S-, G2/M-phases of *Ctf4-GFP* (*n* = 50) and *Ctf4-GFP;dia2*Δ (*n* = 50). The two-tailed Wilcoxon–Mann–Whitney test was used to evaluate statistical significance; ** *p* < 0.001; NS (non-significant) *p* > 0.01.

To study how Dia2 influences the cell cycle in S. cerevisiae, we constructed a Ctf4-GFP strain in *dia2*∆ background (addressed in this study as *Ctf4-GFP;dia2*Δ strain). To ensure that the presence of the GFP tag does not alter the properties and functions of the studied protein, we compared the viability of the *Ctf4-GFP* strain to that of wild-type cells. When grown on a YPD-rich medium, the GFP-tagged strain exhibited viability similar to the wild-type control and an *Mrc1-GFP* strain [28] (Figure 1a). The GFP-tagged strains also showed an ability to withstand chronic exposure to increasing concentrations of the S-phase checkpoint-inducing agent hydroxyurea (HU) [28], similarly to that of wild-type cells (Figure 1a). However, the *dia2*Δ strains showed slightly reduced viability in a YPD medium, which became more pronounced in YPD supplemented with HU (Figure 1a) [23].

To evaluate the overall effect of Dia2 deficiency on cell cycle progression, we measured the duration of the cell cycle by live-cell imaging of *Ctf4-GFP* and *Ctf4-GFP;dia2*Δ strains. To determine the different phases of the cell cycle, we refer to the specific yeast cell and nuclear morphology, taking into account the nuclear localization of Ctf4-GFP. We define the G1-to-S transition as the moment when the bud emerges (Figure 1b,c and Videos S1 and S2), the S-to-G2/M transition when the nucleus enters the bud, and the G2/M-to-G1 transition when there is no visible GFP bridge between the two nuclei and they undergo fast movement in opposite directions, indicating that nuclear division has finished and the cells have separated [29,30]. Live-cell imaging demonstrated that the duration of the cell cycle in *dia2*Δ cells, defined as the interval between two sequential nuclear divisions, was 145 ± 30 min (*n* = 50), which is significantly longer compared to that of the Ctf4-GFP WT cells (95 ± 16 min, *n* = 50) (Figure 1d) [21,31]. The prolonged cell cycle in *dia2*Δ cells was due to the extended S- and G2/M-phases, which were 82 ± 28 and 29 ± 13 min (*n* = 50), respectively, compared to 48 ± 15 and 20 ± 3 min (*n* = 50) in the strain with intact Dia2 (Figure 1e–g) [30]. Our results show that loss of Dia2 increases the duration of the cell cycle due to prolonged S- and G2/M-phases.

### 2.2. Deletion of DIA2 Leads to Increased Levels of Ctf4

**Figure 2:**
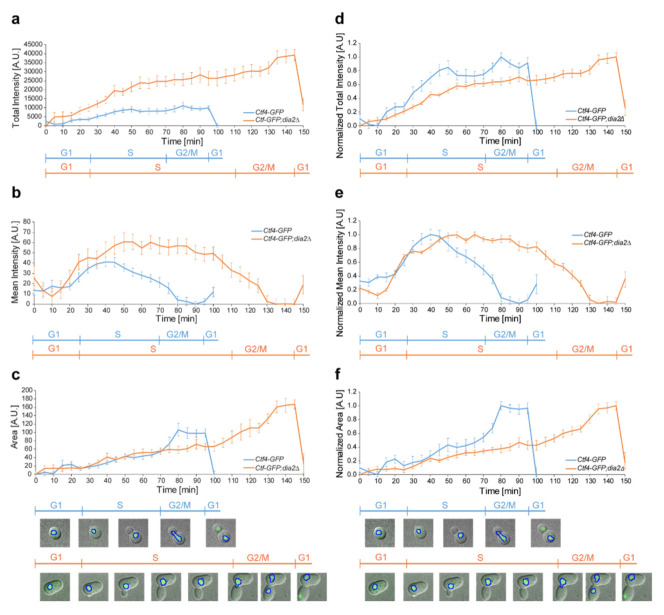
Quantitative and normalized profiles of *Ctf4-GFP* and *Ctf4-GFP;dia2*Δ throughout the cell cycle. (**a**) Averaged total fluorescence intensity (mean intensity × area) of *Ctf4-GFP* and *Ctf4-GFP;dia2*Δ. (**b**) Averaged mean fluorescent intensity of the emitted GFP signal from the nucleus of *Ctf4-GFP* and *Ctf4-GFP; dia2*Δ. (**c**) Averaged area of the nucleus of *Ctf4-GFP* and *Ctf4-GFP;dia2*Δ yeast cells. For (**a**–**c**), the minimum value for each graphic is normalized to 0. (**d**–**f**) The data from (**a**–**c**) are normalized between 0 to 1 and are represented in (**d**–**f**), respectively. For each time point, the background was subtracted and maximum intensity projection function of 11 z-stacks was applied. A total of 10 cells with averaged durations of the cell cycle for each strain were selected and the standard error of mean is represented. Representative images of the two strains demonstrate the measured region of interest (ROI).

It is known that Dia2 interacts with Ctf4 and is responsible for its ubiquitination during DNA replication [25]. This raises the question whether Dia2 deficiency alters the amount of Ctf4 throughout the extended cell cycle. Therefore, we established the quantitative profile of the Ctf4 protein from the *Ctf4-GFP* and *Ctf4-GFP;dia2*Δ strains throughout the entire cell cycle via live-cell microscopy. In order to measure an averaged profile of Ctf4-GFP without data interpolation, we selected cells with cell cycle durations equal to the average for each strain (Figure 2). Since Ctf4 is fused with GFP, we quantified the fluorescence intensity, which is proportional to the amount of endogenous Ctf4. With the progression through the cell cycle, the total amount of Ctf4 in the nucleus increases more rapidly in the deletion strain, reaching a maximum of more than three times at the end of the G2/M-phase compared to the *Ctf4-GFP* strain (Figure 2a). One of the reasons behind these results is the difference in the enlargement of the nuclear area in these two strains (Figure 2c). In the *dia2*Δ cells, the nucleus continues to grow during the extended S- and G2/M-phases, leading to an approximately two-fold increase of its nuclear area compared to the wild-type cells (Figure 2c). In addition, the deletion of DIA2 also increases Ctf4 concentration (mean fluorescence intensity of the nucleus) by approximately 30% during the prolonged S-phase, contributing to the total increase of the Ctf4 amount (Figure 2b). These two reasons elucidate why the total amount of Ctf4 in the *Ctf4-GFP;dia2*Δ strain is considerably increased, compared to the levels observed in the *Ctf4-GFP* strain.

To properly compare the dynamic changes in the amounts of Ctf4 between the two strains throughout the cell cycle, we normalized the data from 0 to 1 (Figure 2d–f). In the strain with intact DIA2 the Ctf4 concentration starts to decrease in the middle of the S-phase, reaching a minimum in the G2/M-phase (Figure 2e). In contrast, Ctf4 levels in the deletion strain start to decrease at the end of the prolonged S-phase. Collectively, our results suggest that the total amount of Ctf4 in *dia2*Δ is higher, and it decreases considerably later in the prolonged S-phase compared to the levels in the *Ctf4-GFP* strain.

**Figure 3:**
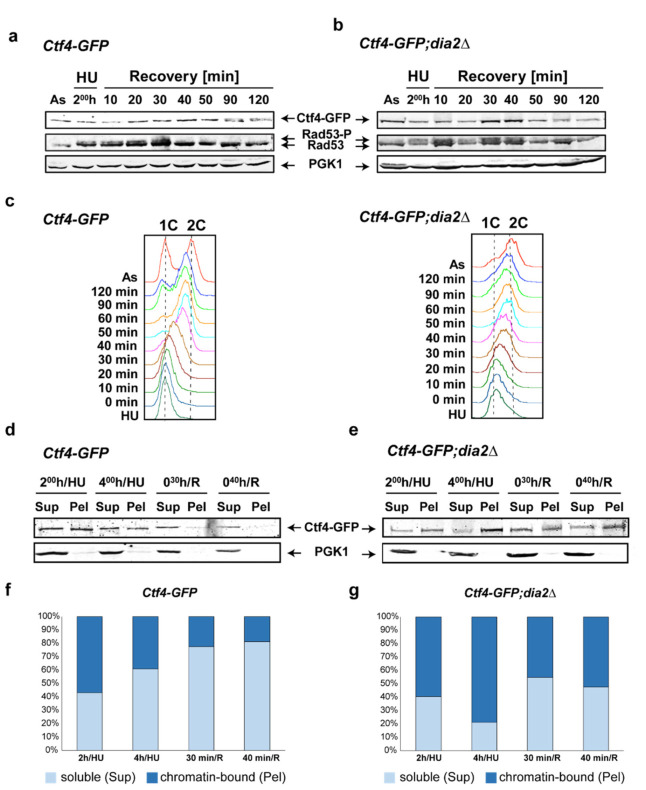
Western blot analysis of Ctf4 profile. (**a**,**b**) Immuno-detection of Ctf4-GFP, Rad53, and PGK1 (loading control) was carried out from total protein extracts obtained at the indicated time points from both *Ctf4-GFP* and *Ctf4-GFP;dia2*Δ strains. Rad53-P and Rad53 indicate the phosphorylated and unphosphorylated forms of Rad53 protein, respectively. The presence of Rad53-P indicates the activation of the S-phase checkpoint. (**c**) Flow-cytometry analysis at the indicated time points for both strains. (**d**,**e**). Chromatin fractionation assay of *Ctf4-GFP* and *Ctf4-GFP;dia2*Δ strains. Samples from the indicated time points were separated into chromatin-bound (Pel) and unbound/soluble (Sup) fractions. The cytoplasmic protein PGK1 was used as an internal loading control. (**f**,**g**) Relative amounts of chromatin-bound (Pel) and soluble (Sup) Ctf4-GFP from the two strains. For each time point, the ratio of the Sup to the Pel is represented; As—asynchronous cell culture; HU—hydroxyurea-arrested cells; R—recovery time point, Sup—Supernatant; Pel—Pellet.

To further shed light on the changes in Ctf4 levels, we performed immunoblot analysis following release from HU replication arrest [32]. Samples were taken at different time points after the release from the HU block, and total protein extracts were isolated, starting from the same number of cells (Figure 3a,b). Flow cytometry analysis was also carried out to determine the cell cycle phase distribution of the cells in each sample (Figure 3c). In agreement with the live-cell imaging experiments, a higher amount of Ctf4-GFP was detected in the samples from 30 and 40 min following release from HU arrest, when the DIA2 gene was deleted (Figure 3a,b). These time points correspond to the end of S-phase and the beginning of G2/M-phase as demonstrated by flow-cytometry analysis (Figure 3c). Interestingly, the deactivation of the S-phase checkpoint after release from HU arrest is delayed in the *dia2*Δ strain. First, the HU treatment activated the S-phase checkpoint, as indicated by the presence of the hyperphosphorylated form of the Rad53 kinase [33] (Figure 3a,b). Subsequently, the S-phase checkpoint was gradually attenuated, indicated by Rad53 dephosphorylation, which completely disappeared 40 min following HU removal (Figure 3a). In addition, phosphorylated Rad53 is observed even without HU treatment when DIA2 is missing, which indicates that Dia2 loss, per se, can activate the cell cycle checkpoints (Figure 3b).

### 2.3. Dia2 Deficiency Increases the Chromatin-Bound Fraction of Ctf4

To investigate whether the change in Ctf4 levels involves chromatin-bound Ctf4, we performed a bulk chromatin fractionation assay. Crude soluble supernatant (Sup) and chromatin pellet (Pel) fractions were isolated from wild-type and *dia2*Δ strains at the corresponding time points and were analyzed via immunodetection, which allowed us to discriminate between chromatin-bound and soluble fractions of Ctf4-GFP (Figure 3d,e). The protein was bound to the chromatin in both strains 2 h following HU treatment. This suggests that while DNA synthesis is perturbed and the S-phase checkpoint is active, Ctf4 is bound to the stabilized replisomes [34] (Figure 3d,e). However, the levels of chromatin-bound Ctf4 gradually diminished in the *Ctf4-GFP* strain 30 min following release from HU arrest (Figure 3d,e). In contrast, in the *Ctf4-GFP;dia2*Δ strain, the reduction of chromatin-bound Ctf4-GFP at the same time points was negligible, implying a role for Dia2 in Ctf4 destabilization during checkpoint recovery (Figure 3f,g). This is in agreement with the fact that Ctf4 is ubiquitinated in a Dia2-dependent manner in the course of DNA replication termination [25]. Interestingly, the prolonged HU treatment of cells with intact DIA2 for up to 4 h decreased the amount of chromatin-bound Ctf4, compared to the 2 h-treatment (Figure 3d,f). These results confirm that, in these conditions, the cells manage to adapt to diminished nucleotide levels and proceed with DNA synthesis, albeit at a low speed [28] (Figure 5c). Over time, a fraction of the cells complete DNA replication, reducing the level of chromatin-bound Ctf4 (Figure 3d,f). Notably, prolonged treatment with HU (4 h) led to an increased level of chromatin-associated Ctf4 in *dia2*Δ yeast cells (Figure 3e,g). Collectively, our results show that *dia2*Δ increases the amount of chromatin-bound Ctf4 at the end of the S-phase/ beginning of the G2/M-phase and during checkpoint adaptation, where cells overcome the S-phase checkpoint arrest even when the factors perturbing replication-fork progression are still present [28].

### 2.4. Lack of Dia2 Leads to Abnormal Cell Size

**Figure 4:**
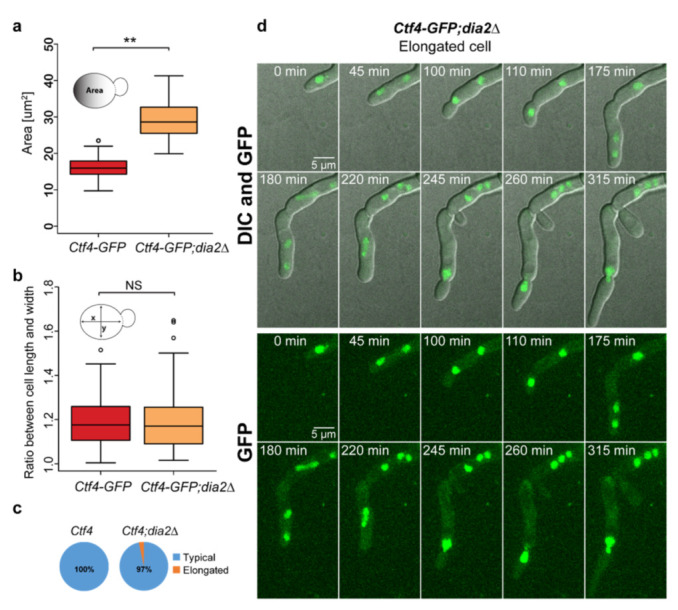
Deletion of DIA2 leads to abnormal cell size and morphology. (**a**) Area of the cells from Ctf4-GFP (*n* = 90) and *Ctf4-GFP;dia2*Δ (*n* = 90). (**b**) Long-to-short (x-to-y) cell axis ratio of Ctf4-GFP (*n* = 90) and *Ctf4-GFP;dia2*Δ (*n* = 90). (**c**) Proportions of elongated and typical cells. (**d**) An example of the elongated and multinucleated *Ctf4-GFP;dia2*Δ group of cells. Maximum Intensity Projection of 11 z-planes was applied. The two-tailed Wilcoxon-Mann-Whitney test was used to evaluate statistical significance; ** *p* < 0.001; NS (non-significant) *p* > 0.01 and mean value ± standard deviation was used.

Interestingly, live-cell imaging revealed a morphological difference between cells from *Ctf4-GFP* (Figure 1b, Video S1) and *Ctf4-GFP;dia2*Δ strains (Figure 1c and Figure 4d, Videos S2 and S3). We observed that DIA2 deletion led to a 1.8-fold increase in cell size (Figure 4a). Cells from the *Ctf4-GFP* strain exhibited an average area of 16.15 ± 2.78 µm^2^ (*n* = 90), whereas cells with missing Dia2 had an average area of 28.95 ± 5.08 µm^2^ (*n* = 90). It is known that mutations that prolong the cell cycle concomitantly increase the cell size in yeast [35]; therefore, we have assumed that the prolonged cell cycle of *dia2*Δ cells leads to an increase in cell size. Despite the large size of the cells, we do not observe a significant difference in the ratio of the long-to-short (x-to-y) axes (Figure 4b). The average ratio of the long-to-short (x-to-y) axes in both strains is 1.19 (1.19 ± 0.11 for *Ctf4-GFP* and 1.19 ± 0.14 for *Ctf4-GFP;dia2*Δ, *n* = 90). However, 3% of the *dia2*Δ cells exhibited an elongated phenotype (a ratio of the long-to-short axes is above 1.6) that we do not observe in the wild-type cells (Figure 4c). It is known that several S. cerevisiae strains are capable of filament-like growth under starvation conditions [27] and that deletion of DIA2 in the Σ1278b strain promotes mild pseudohyphal phenotype [20,26]. However, our results revealed that *dia2*Δ caused pronounced cell elongation even in the filamentation-deficient haploid strain that we used. This strain is derived from the S288C background and lacks Flo8, a key component for filamentous growth [20,27,36,37] (Figure 4d). Notably, we also found that the elongated *dia2*Δ cells were frequently multinucleated (Figure 4d and Video S3). This is not typical for the pseudohyphal phenotype, where cells are elongated but mononucleated and fully separated by cytokinesis [27]

### 2.5. Inhibition of DNA Synthesis Enhances the Oversized Phenotype in dia2Δ Cells

**Figure 5:**
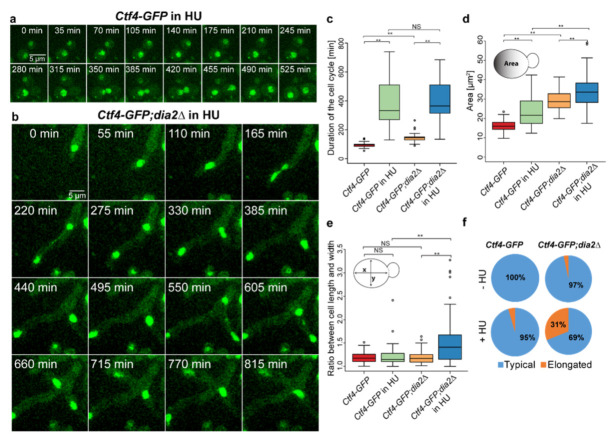
Effect of DNA synthesis inhibition on cell size and morphology. (**a**,**b**) Time-lapse live-cell imaging of *Ctf4-GFP* and *Ctf4-GFP;dia2*Δ yeast cells in HU, respectively. (**c**) Duration of the cell cycle with and without HU (*n* = 50). (**d**) Area of the cells (*n* = 90) from *Ctf4-GFP* and *Ctf4-GFP;dia2*Δ with and without HU treatment. (**е**) Long-to-short (x-to-y) axis ratio (*n* = 90) of *Ctf4-GFP* and *Ctf4-GFP;dia2*Δ with and without HU treatment. (**f**) Proportions of elongated and typical cells of *Ctf4-GFP* and *Ctf4-GFP;dia2*Δ with and without HU treatment. The two-tailed Wilcoxon–Mann–Whitney test was used to evaluate statistical significance; ** *p* < 0.001; NS (non-significant) *p* > 0.01 and mean value ± standard deviation was used.

To reveal whether cell cycle perturbation contributes to the increased cell size, we treated cells with HU, which significantly delays replication progression [32]. Time-lapse experiments of the strain with intact Dia2 (Figure 5a and Video S4) revealed that stalling DNA synthesis by HU tremendously delayed the cell cycle (from 95 ± 16, *n* = 50 to 385 ± 155 min, *n* = 50) (Figure 5c). In addition, wild-type cells managed to divide while in HU, but this was accompanied by a marked increase in cell size, from 16.15 ±2.8 µm^2^ to 23.4 ± 7.78 µm^2^ (Figure 5d). Furthermore, cells with intact DIA2 do not exhibit elongated phenotype when treated with HU (long-to-short cell axis ratio of 1.16 ± 0.12, *n* = 90) (Figure 5e). As shown before, our results confirm that the delayed cell cycle, induced by DNA synthesis inhibition, increases the cell size [38]. Similarly, HU treatment of *dia2*Δ cells significantly increased the cell cycle duration from 145 ± 30 min to 415 ± 147 min (Figure 5b,c and Video S5). This also led to an overall cell size increase from 28.95 ± 5.08µm^2^ in non-treated cells (*n* = 90) to 34.24 ± 8.14µm^2^ in HU treated *dia2*Δ cells (*n* = 90). Our data indicates that the additional increase of cell size when the cell cycle is prolonged by HU treatment confirms that extending the cell cycle has a role in cell size alteration (Figure 5d). Interestingly, while the duration of the cell cycle of the HU-treated wild-type cells is much longer than the duration of the non-treated *dia2*Δ cells, the cell size of the wild-type cells is smaller. These results indicate that *dia2*Δ could influence the cell size not only by prolonging the cell cycle. We also observed that the long-to-short cell axis ratio increased from 1.19 ± 0.14 (*n* = 90) to 1.52 ± 0.50 (*n* = 90) in *dia2*Δ cells when treated with HU (Figure 5f). Additionally, HU also increased the proportion of elongated cells 10-fold in the *dia2*Δ strain compared to non-treated *dia2*Δcells (Figure 5f), indicating that perturbed DNA replication promotes the elongated phenotype when Dia2 is missing.

Collectively, we show that the deletion of DIA2 leads to cells with increased cell size and extended S- and G2/M-phases with elevated levels of Ctf4. In addition, a comparison of the effects of DNA replication inhibition and of the absence of Dia2 on cell cycle duration and cell size suggests that the increased cell size in *dia2*Δ involves a complex mechanism for which the extended cell cycle is only one of the triggers.

## 3. Discussion

In this study, we investigated the effects of Dia2 deficiency on the cell cycle, cell size, and Ctf4 recruitment in *S. cerevisiae* cells. It is known that in budding yeast, Dia2 is responsible for maintaining genomic stability and plays a key role in DNA replication. Its levels are low during the G1-phase and increase during the S-phase, corresponding to its function [19,39]. In this study, we showed that *dia2*Δ cells exhibit an extended cell cycle duration due to prolonged S- and G2/M-phases, which also suggests a role of Dia2 in DNA replication and S-phase checkpoint control (Figure 1) [20,21]. As a control mechanism, the S-phase checkpoint activates a cascade of events that blocks DNA synthesis, stabilizes replication forks, and suppresses late origin firing [40,41,42,43].

Interestingly, we, as well as other groups [20,44], observed Rad53 phosphorylation in the asynchronous sample when Dia2 is missing, which is indicative of perturbed DNA replication (Figure 3a,b). Moreover, following release from DNA synthesis arrest, Rad53 dephosphorylation is delayed, suggesting a slower recovery rate in *dia2*Δ cells. Rad53 plays an essential role in the S-checkpoint as an effector kinase. It functions to phosphorylate downstream proteins, thus keeping the replisome intact and ensuring that the cell will have enough time to remove the obstacles for faithful DNA replication [45,46,47]. Therefore, the prolonged Rad53-dependent checkpoint could lead to the extended S- and G2/M-phases, which we detected in the *dia2*Δ strain (Figure 1d,f,g).

To shed light on how Dia2 deficiency leads to extended S- and G2/M-phases, we studied the level of Ctf4 throughout the cell cycle. It has been shown that Dia2, as part of the SCF^Dia2^ E3 ubiquitin ligase complex, associates with replication forks via a direct physical interaction with Mrc1 and Ctf4 [23]. Here, we show that the levels of Ctf4, a binding partner of Dia2, are higher at the end of the extended S- and G2/M-phases in the absence of functional SCF^Dia2^ compared to the strain where DIA2 is present (Figure 2). Furthermore, we demonstrated that, in *dia2*Δ cells, Ctf4 levels are elevated in the time interval of 30 to 40 min following recovery from hydroxyurea treatment (Figure 3a,b). We demonstrated that the fraction of chromatin-bound Ctf4 is also increased at these time points in *dia2*Δ cells (Figure 3d–g). The elevated levels of Ctf4 might be a result of reduced degradation by the SCF^Dia2^ ubiquitin ligase. This is supported by the fact that Dia2 ubiquitinates Ctf4 in vitro in the course of DNA replication termination [25]. In addition, it is known that SCF^Dia2^ is responsible for MCM7 ubiquitination, leading to the disassembly of the active helicase at the end of the S-phase [48]. As Ctf4 binds to GINS, a component of the CMG helicase, another possible explanation for the high level of chromatin-bound Ctf4 could be the reduced Dia2-dependent disassembly of the CMG complex at the end of DNA replication. Perturbed CMG disassembly and the termination of DNA replication in *dia2*Δ cells could lead to the activation of the checkpoint in some cells and extend the S- and G2/M-phases of the cell cycle. It is known that Ctf4 functions as a hub that coordinates the Polα/Primase complex and the CMG helicase [12,13]. Interestingly, such as for Mrc1 [26], we found that a prolonged incubation with HU led to decreased levels of chromatin-bound Ctf4 when DIA2 was intact (Figure 3d,f). This could be a consequence of the adaptation process to HU, during which cells overcome the S-phase checkpoint arrest even when the factors perturbing replication-fork progression are still present [28]. In contrast, when Dia2 is missing, we observed a higher amount of chromatin-bound Ctf4 during continuous incubation with HU (Figure 3e,g). Our results suggest that, similarly to the recovery from replication arrest, the prolonged incubation in HU leads to an increase in chromatin-bound Ctf4 in *dia2*Δ cells.

In addition to the role of Dia2 in DNA replication, we found that *dia2*Δ cells exhibit increased cell size (Figure 4a,d). However, the contribution of Dia2 to this process is still unclear. Hartwell et al. showed that mutations in some Cdc proteins and inhibitors, which arrest cells at different stages of the cell cycle, cause the formation of cells with increased cell size [35]. In our yeast background, we also observed that wild-type cells, when treated with HU that prolongs the cell cycle, display increased cell size (Figure 5d). Therefore, it is expected that the extension of the cell cycle in *dia2*Δ cells increases the cell size. However, Dia2 deficiency leads to a significantly lesser effect on cell cycle duration, but a much more profound increase in cell size compared to DNA replication inhibition (Figure 5d,e). Hence, prolonging the cell cycle, per se, cannot explain the substantial increase in the cell size of *dia2*Δ cells. This emphasizes the multi-layered role of Dia2 for cell size regulation. We found that the deletion of Dia2 has little effect on cell elongation (Figure 5e). However, the inhibition of DNA replication in *dia2*Δ cells significantly promotes cell elongation in 30% of the cells, even in the strain that we used, which is deficient for the essential factor for preudohypal growth (Figure 5e,f). This suggests that DIA2 deletion predisposes cells to cell elongation, but other factors, such as DNA replication perturbations, are required for the cells to reveal such a phenotype.

Our study demonstrates that, in *Saccharomyces cerevisiae*, Dia2 deficiency extends the S- and G2/M-phases of the cell cycle and activates the S-phase checkpoint. A lack of Dia2, which is part of SCF^Dia2^ E3 ubiquitin ligase, stabilizes the chromatin-bound form of Ctf4 during the elongated S- and G2/M-phases. The prolonged cell cycle in the absence of Dia2 is accompanied by a marked increase in cell size; however, the extended cell cycle alone is not enough to elucidate the increase of cell size in the *dia2*Δ background, suggesting a complex control mechanism where Dia2 is a key player.

## 4. Materials and Methods

### 4.1. Strains

*Ctf4-GFP* (MATa his3Δ1 leu2Δ0 met15Δ0 ura3Δ0 CTF4-GFP-HIS3MX6) and *Mrc1-GFP* (MATa his3Δ1 leu2Δ0 met15Δ0 ura3Δ0 MRC1-GFP-His3MX6) (used as an additional control) [49] are, with the BY 4741 background, derived from S288C, and were obtained from Invitrogen™. These strains were transformed with the corresponding cassette [50] to create strains in which the DIA2 gene is deleted. We used the plasmid pUG6 (4009 bp) for PCR amplification with specific primers (Table 1) of these disruption cassettes, which contain a selection marker KanMX for Geneticin (G418) resistance and 50 bp flanking sequences (introduced by the PCR primers) homologous to regions of the target genes. Selection, with 200-μmol/mL G418, and diagnostic PCR was performed to confirm the integration of the disruption cassettes [51]. The pairs of diagnostic primers used were designed so that one of them is complementary to the yeast genome region neighboring the integrated cassette, and the other to the sequence from the KanMX gene (Table 1). In this way, *Ctf4-GFP;dia2*Δ (MATa his3Δ1 leu2Δ0 met15Δ0 ura3Δ0 CTF4-GFP-His3MX6 dia2Δ::KanMX) and *Mrc1-GFP;dia2*Δ (MATa his3Δ1 leu2Δ0 met15Δ0 ura3Δ0 MRC1-GFP-HIS3MX6 dia2Δ::KanMX) strains were created. All strains were cultivated in YPD medium (1% (*w*/*v*) yeast extract (Difco), 2% (*w*/*v*) Bacto peptone (Difco), 2% (*w*/*v*) dextrose). As a control, a wild-type (WT) S288C strain without a GFP tag was used.

### 4.2. Viability Test

To perform the 10-fold serial dilution assays, yeast samples were prepared from exponentially growing cultures at a concentration of 3.4 × 10^6^ cells/mL. Five μL of each dilution were then spotted onto YPD, or YPD supplemented with 50 mM, 100 mM, or 200 mM HU. Plates were incubated at 25 °C for three days.

### 4.3. Time-Lapse Live-Cell Imaging of Yeast Cells

In order to reduce autofluorescence, prior to live-cell imaging, yeast cells were pre-incubated in CSM media (1.7 g/L YNB, 0.04 g/L CSM-His and 2% (*w*/*v*) dextrose) with 20 μg/mL extra adenine (Ade) until OD_600_ = 0.2. A total of 1 mL yeast suspension was centrifuged at 4000 rpm for 1 min, and the pellet was resuspended in 50 μL of fresh CSM with Ade. Prior to microscopy, a concavity microscope glass slide was prepared. The slide contained a cavity in the center, which was filled with CSM and agarose (1.2%), and 2.5 μL of the cell suspension was placed on it. The edge of the hole was greased with Vaseline to create better adhesion between the coverslip and the slide, as well as to reduce deformation of the media for microscopy inside the cavity. The slide was then covered with a cover glass, and slight pressure was applied. All procedures were carried out at 27 °C and according to the protocol described by Silva and co-workers [52]. For some of the experiments, HU was added to the solid and liquid media.

Time-lapse experiments were performed using a Yokogawa CSU-X1 spinning disc confocal microscope (Andor Revolution XD system with a Nikon TiE microscope stand and an incubator for temperature and humidity control) equipped with an iXon897 EMCCD camera and a CFI Apo TIRF 100X Oil 1.49 NA objective. All experiments were carried out under the following parameters: 11 Z-planes, with a Z-step of 0.5 μm; laser intensity: 8.1% on 488 nm; camera exposure of 200 ms. Frames were taken every 5 min for the whole duration of the experiments, and the maximum intensity projection function was applied to all the acquired images. All data were analyzed using ImageJ and CellTool/Version 1.6.0.2 (http://dnarepair.bas.bg/software/CellTool/, accessed on 17 July 2021) [53] software. The average intensity and the area of the emitted GFP signal in the nucleus of the yeast cells were measured for each frame. Background fluorescence was subtracted from all images. For Figure 2, 10 cells were selected per strain with cell cycle duration as the average: 95 min for *Ctf4-GFP* and 145 min for *Ctf4-GFP;dia2*Δ, and the standard error of the mean is shown as error bars. Normalization was carried out, taking the minimum value as 0 and the maximum as 1. Live-cell imaging from a minimum of 3 experiments per strain was also used to determine the duration and the phases of the cell cycle based on characteristic cell and nuclear morphology [29,30]. The ratio of the long axis length (x) to the short axis length (y) of 90 cells was measured for each strain. If the ratio was under or equal to 1.6, the cells were considered “Typical”. Alternatively, if the ratio was above 1.6, the cells were considered “Elongated”. For the area and ratio measurements, cells with nuclear and cellular morphology specific to budded S-phase cells were chosen without including the bud in the measurement. For all box plots, the two-tailed Wilcoxon–Mann–Whitney test was used to evaluate the statistical significance (* *p* < 0.001; 0.001 < *p* < 0.01; NS *p* > 0.01) and the standard deviation was presented as error bars.

### 4.4. Bulk Chromatin Fractionation

In order to prepare chromatin-bound and soluble fractions, we used a previously described protocol [54] with some modifications. 0.1% NaN_3_ was added to 1 × 10^9^ harvested cells treated with 100 mM HU and were incubated for 30 °C. Then, the cells were treated with 3 mL of prespheroplasting buffer (100 mM PIPES (pH 9.4), 10 mM DTT) for 10 min, followed by incubation in 1 mL spheroplasting buffer (50 mM KH_2_PO_4_/K_2_HPO_4_ (pH 7.5), 0.6 M Sorbitol, 10 mM DTT) for 5 min. Following LongLife™ Zymolyase^®^ (Geno Technology, Inc., Saint Louis, MO, USA; Cat. # 786–036) digestion, the spheroplast pellets were washed with 1 mL of ice-cold wash buffer (100 mM KCl, 50 mM HEPES KOH (pH 7.5), 2.5 mM MgCl_2_, and 0.4 M Sorbitol). Next, the cells were pelleted at 4000 rpm for 1 min at 4 °C. The pellets were measured and resuspended in an equal volume of extraction buffer (EB; 100 mM KCl, 50 mM HEPES-KOH (pH 7.5), 2.5 mM MgCl_2_, 50 mM NaF, 5 mM Na_4_P_2_O_7_, 0.1 mM NaVO_3_) with 1.5% Triton X-100, 1 mM PMSF, and protease inhibitors cocktail (Complete Mini EDTA-free Protease Inhibitor Cocktail Tablets, # 05892791001, Roche, Basel, Switzerland) followed by incubation for 10 min at 4 °C. To remove the aggregated and unlysed cells from the suspension, the lysates were passed through a thin syringe needle and spun at 300 g. Then, the lysates were then under-layered with 30% sucrose and spun at 12,000 rpm for 10 min at 4 °C. The pellet (chromatin-bound) fractions were washed with 25% volume of the EB containing 1.5% Triton X-100 (EBX) and spun again at 10,000 rpm for 5 min at 4 °C. The crude chromatin pellets were dissolved in EBX. The supernatants (Sup) served as a soluble, chromatin-unbound fraction. Finally, the volumes of Sup and Pel were equalized with EBX, and 2× Laemmli buffer was added to each fraction. Samples were boiled for 3 min and spun at 10,000 rpm for 1 min before loading on 6–15% gradient SDS PAGE gels.

### 4.5. Total Protein Extraction

For the recovery experiments, cells were first treated with HU for 2 h, and HU was then washed. Total protein extracts from 6 × 10^8^ yeast cells were prepared for each time point by TCA precipitation, as described by Foiani and co-workers [55]. All solutions were supplemented with a protease inhibitor cocktail (cOmplete Mini EDTA-free Protease Inhibitor Cocktail Tablets, # 05892791001, Roche) and the phosphatase inhibitors 0.1 mM Na_3_VO_4_ and 1 mM NaF.

### 4.6. Western Blotting

The samples were loaded on 6–15% gradient SDS-PAGE gels, ran at 140 V, and then transferred onto a Protran blotting nitrocellulose membrane (Cat # 10600003). For detection of the Ctf4 protein, a goat polyclonal Anti-EGFP antibody was used (kindly provided by Max Planck Institute for Molecular Cell Biology and Genetics [56]). The results were visualized by an Odyssey Infrared Imaging system (Li-Cor) via IR Dye 680RD Donkey Anti-Goat Antibody (#926-68074, Li-Cor). To detect Rad53, we used a goat polyclonal anti-Rad53 antibody (Rad53 y-19 from Santa Cruz Biotechnology, Santa Cruz, CA, USA) and the IR Dye 680RD Donkey Anti-Goat Antibody (# 926–68074, Li-Cor). PGK1 was immunodetected by mouse monoclonal anti-PGK1 antibody (22C5D8) (ab113687, Abcam, Cambridge, UK) and an IR Dye 800CW Goat Anti-Mouse Antibody (#926-32210, Li-Cor, NE, USA). Original images for the blots are available as Supplementary Figure S1.

### 4.7. Flow Cytometry Analysis

Flow cytometry was used to determine the amount of DNA in each cell by measuring its fluorescence signal (λ488), emitted from the intercalated propidium iodide. The yeast cells were pelleted and then fixed using 70% ethanol, followed by resuspension in 0.28 М Tris-HCl (pH 7.5) and sonication for 10 s using a power of 30 V (to disrupt cell adhesion). A total of 2 mg/mL RNAse А was added to each sample, and cells were incubated for 12 h at 37 °C. Following incubation, cells were centrifuged, and the pellet was resuspended in 180 mM Tris-HCl pH 7.5 (190 mM NaCl and 70 mМ MgCl_2_). Finally, the cells were incubated with propidium iodide (50 μg/mL), diluted in 50 mM Tris-HCl (pH 7.8), and analyzed using Becton Dickinson FACScan.

## Figures and Tables

**Table 1:** PCR primers used in this study.

Primer	Application	Sequence
Dia2-disr UP pUG6	Gene disruption	TTCTCGAAAAATATTATAAATAGACATGCAAAATGATTAGCCATGCAGCTGAAGCTTCGTACGC
Dia2-disr DOWN pUG6	Gene disruption	ATTTTCCGAAGGATACTGCATTATCATCAGTGATTTATTAATCTAGCATAGGCCACTAGTGGATCTG
Dia2-check 5′_UP	Diagnostic PCR	CGGCAATCTTCACACGGT
Dia2-check 3′_DOWN	Diagnostic PCR	GTTTGCCATCGGTGCATC

## Data Availability

Not applicable.

## Supplementary Materials

The following are available online. Video S1. Live-cell imaging of *Ctf4-GFP* yeast cells throughout the entire cell cycle.; Video S2. Live-cell imaging of *Ctf4-GFP;dia2*Δ yeast cells throughout the entire cell cycle.; Video S3. Live-cell imaging of *Ctf4-GFP;dia2*Δ elongated cell.; Video S4. Live-cell imaging of *Ctf4-GFP* yeast cells throughout the cell cycle in the presence of HU.; Video S5. Live-cell imaging of *Ctf4-GFP;dia2*Δ yeast cells throughout the cell cycle in the presence of HU.; Figure S1. Original images for blots.

## Sample Availability

The strains used in this study are available upon reasonable request.
